# Evaluation of hepatic integrin αvβ3 expression in non-alcoholic steatohepatitis (NASH) model mouse by ^18^F-FPP-RGD_2_ PET

**DOI:** 10.1186/s13550-018-0394-4

**Published:** 2018-05-31

**Authors:** Takemi Rokugawa, Haruyo Konishi, Miwa Ito, Hitoshi Iimori, Ryohei Nagai, Eku Shimosegawa, Jun Hatazawa, Kohji Abe

**Affiliations:** 1 Translational Research Unit, Biomarker R&D Department, Shionogi & Co., Ltd., 3-1-1, Futaba-cho, Toyonaka, Osaka, 561-0825 Japan; 2 Obesity and Metabolic Diseases, Drug Discovery and Disease Research Laboratory, Shionogi & Co., Ltd., Osaka, Japan; 3 Department of Applied Chemistry and Analysis, Research Laboratory for Development, Shionogi & Co., Ltd., Osaka, Japan; 4 Department of Molecular Imaging in Medicine, Osaka University Graduate School of Medicine, Osaka, Japan; 5 Department of Nuclear Medicine and Tracer Kinetics, Osaka University Graduate School of Medicine, Osaka, Japan; 6 PET Molecular Imaging Center, Osaka University Graduate School of Medicine, Osaka, Japan

**Keywords:** Non-alcoholic fatty liver disease, Non-alcoholic steatohepatitis, Fibrosis, Positron emission tomography, ^18^F-FPP-RGD_2_, Modified methionine choline-deficient, High-fat diet, Integrin αvβ3

## Abstract

**Background:**

Activated hepatic stellate cells (HSCs), which express integrin αvβ3, are a major fibrogenic factor in NASH pathophysiology. ^18^F-labeled cyclic arginine-glycine-aspartic acid penta-peptide (^18^F-FPP-RGD_2_) has been used as a PET probe for tumors expressing integrin αvβ3. The aim of this study was to assess the potential of PET with ^18^F-FPP-RGD_2_ to detect hepatic integrin αvβ3 expression in non-alcoholic steatohepatitis (NASH) model mice.

**Results:**

Thirty-two male C57BL/6 mice aged 6 weeks were fed a choline-deficient, l-amino acid-defined, high-fat diet (CDAHFD) for 3 and 8 weeks. ^18^F-FPP-RGD_2_ PET imaging of the liver was performed at 3 and 8 weeks after CDAHFD feeding. After PET scanning, levels of hepatic integrin αvβ, 3α-smooth muscle actin (α-SMA), and collagen type 1 alpha 1(col1a1) were measured. Histopathological analysis of hepatic steatosis, inflammation, and fibrosis, as well as blood biochemistry analysis, was also performed. CDAHFD for 3 and 8 weeks produced a moderate-to-severe steatosis and inflammation of the liver in mice. NAFLD activity score (NAS) in mice fed the CDAHFD for 3 and 8 weeks were more than 4 indicating NASH or borderline NASH pathology. Fibrosis was observed only in mice fed the CDAHFD for 8 weeks. PET imaging showed that the hepatic standardized uptake value, SUV_80–90 min_, was increased with prolonged CDAHFD feeding compared with the respective controls (CDAHFD 3 weeks 0.32 ± 0.06 vs 0.48 ± 0.05, *p* < 0.01; CDAHFD 8 weeks 0.35 ± 0.04 vs 0.75 ± 0.07, *p* < 0.01, respectively). Prolonged CDAHFD feeding increased hepatic mRNA and protein levels of integrin αv and β3 at 3 and 8 weeks. Hepatic ^18^F-FPP-RGD_2_ uptake and amount of integrin αv and β3 protein were well correlated (*r* = 0.593, *p* < 0.05 and *r* = 0.835, *p* < 0.001, respectively). Hepatic ^18^F-FPP-RGD_2_ uptake also showed a positive correlation with Sirius red-positive area.

**Conclusions:**

The hepatic uptake of ^18^F-FPP-RGD_2_ correlated well with integrin αv and β3 expression and histological fibrosis in a mouse model of NASH, suggesting the predictability of fibrosis in NASH pathology.

## Background

Non-alcoholic fatty liver disease (NAFLD), one of the most common forms of chronic liver disease in patients without a history of alcoholic abuse, encompasses a wide spectrum of conditions from simple steatosis to non-alcoholic steatohepatitis (NASH) [1]. It was reported that 30–40% of NASH patients progress to fibrosis and about 10% progress to cirrhosis [2]. The prognosis of NAFLD depends on the histological severity, particularly of liver fibrosis, which is the strongest predictor of liver morbidity and mortality [3]. To prevent liver-related mortality, it is important to reverse advanced fibrosis or prevent the progression to fibrosis in NASH patients.

From a clinical view point, liver biopsy is the gold standard for the diagnosis of NASH and staging liver fibrosis [4]. However, liver biopsy has limitations including sampling error and it is invasive, painful and results in poor patient compliance [5]. Therefore, the development of a non-invasive strategy to evaluate liver fibrosis is required. Magnetic resonance elastography and ultrasound-based transient elastography have been developed to assess liver fibrosis [6, 7]. These methods can discriminate moderate and advanced liver fibrosis from early-stage liver injury or the normal patient population. However, these approaches have reported a lower accuracy for the detection of early stage liver fibrosis [8]. Furthermore, especially in NASH patients, steatosis may produce a softer liver because of fat deposition in the liver parenchyma [9, 10]. These non-invasive tools are not likely to be sensitive enough to identify mild changes or early stages of fibrosis. Preclinical studies recently reported the imaging of collagen or elastin probes for the reliable assessment of fibrosis [11, 12]. Thus, the development of sensitive imaging markers of fibrogenesis is important to predict the prognosis and determine the precise therapeutic intervention required.

Activated hepatic stellate cells, myofibroblasts, cholangiocytes, macrophages, and components of the pathological extracellular matrix are major fibrogenic factors. Activation and proliferation of hepatic stellate cells (HSCs) are considered key factors of hepatic fibrosis [13]. Following HSC activation, they transform to myofibroblast-like cells characterized by α-smooth muscle actin (α-SMA) expression and produce excessive amounts of extra cellular matrix proteins such as type 1 and type 3 collagen [13]. With the activation of HSCs, integrin αvβ3, which plays an important role in cell signaling such as cell-to-cell adhesion, apoptosis, and cell-matrix interactions, is expressed on HSCs [14]. Thus, monitoring the expression of integrin αvβ3 in NASH liver might be used as a marker to predict the onset of fibrosis.

The three-amino-acid sequence of arginine-glycine-aspartic acid (RGD) has a high binding affinity to integrin αvβ3 [15, 16]. Many studies reported that radiolabeled cyclic RGD peptides (cRGD) imaged by positron emission tomography (PET) and single photon emission computed tomography (SPECT) have been developed as a new radio tracer for selective integrin αvβ3 positive tumors [17, 18]. In a recent fluorescence trace study, cRGD was accumulated in activated but not quiescent HSCs [19]. Furthermore, hepatic integrin αvβ3 imaging by SPECT using ^125^I and ^99m^Tc-labeled cRGD and magnetic resonance imaging using cRGD labeled by contrast agent USPIO detected rodent hepatic fibrosis induced by thioacetamide or CCl_4_ treatment [19, 20]. These hepatic fibrosis models develop fibrosis more quickly than common NASH models. In NASH pathology, HSCs are activated before or at the early stage of hepatic fibrosis [21]. Therefore, RGD imaging might be a useful predictor for the onset of fibrosis in NASH.

In the present study, we investigated the relationship between the hepatic uptake of ^18^F-FPP-RGD_2_ and integrin αvβ3 expression using PET imaging in a NASH mouse model induced by feeding with a choline-deficient, l-amino acid-defined, high-fat diet (CDAHFD) [22].

## Methods

### Animals and experimental design

Male C57BL/6J mice, aged 6 weeks old, were purchased from CLEA Japan (Shizuoka, Japan). Mice were given free access to water and either a normal diet or CDAHFD, which contained 0.1% methionine, no choline and 60 kcal% fat, prepared by Research Diets (New Brunswick, NJ, USA), for 3 and 8 weeks. They were housed in a temperature-controlled room maintained on a 12 h light/dark cycle with lights on at 7:00 am. The experimental protocols were reviewed and approved by the Institutional Animal Care and Use Committee of Osaka University Graduate School of Medicine.

### Biochemical and histopathological analysis

Mice were euthanized by exsanguination under isoflurane anesthesia. Plasma (200 μL) was collected and assayed for the content of alanine aminotransferase (ALT), aspartate aminotransferase (AST), triglyceride (TG), total cholesterol (TC), and high-density lipoprotein cholesterol (HDLC). The left hepatic lobes were fixed in 10% formalin and sectioned, and 4-μm sections were stained with hematoxylin and eosin (H&E) and Sirius red. Steatosis, inflammation, ballooning, and fibrosis in the liver were assessed based on the severity and size of the lesion. Steatosis and inflammation scores ranged from 0 to 3: normal = 0; minimal = 1; moderate = 2; marked = 3. Ballooning score ranged from 0 to 2: normal = 0; minimal = 1; marked = 2. NAFLD activity score (NAS) was calculated by using the sum of each histological score. To assess hepatic fibrosis, five different areas (× 200 magnification) were selected per mouse, and Sirius red-positive areas were measured using WinROOF software (Mitsutani, Tokyo, Japan).

### Radiopharmaceutical preparation

^18^F-FPP-RGD_2_ was radio synthesized using a two-step method as reported previously [23]. Conjugation between ^18^F-4-nitrophenyl-2-fluoropropionate (^18^F-NFP) and the RGD dimeric peptide (PEG_3_-c[RGDyK]_2_) was performed. Radiochemical purity and specific activity were > 99% and 445.6 ± 107.6 GBq/μmol, respectively, with a radiosynthesis and purification time of 90 min.

### PET imaging

PET scan and X-ray CT imaging were performed with a Pre-Clinical Imaging System Triumph LbPET12/CT (TriFoil Imaging Inc., Chatsworth, CA, USA). Mice fed a CDAHFD or control diet for 3 or 8 weeks were anesthetized with 2% isoflurane. Eight mice per group were used for PET imaging. In each group, five mice were used for non-blockade study and three mice were used for blockade study. Under isoflurane anesthesia, a venous catheter was introduced through the tail vein and used for the administration of ^18^F-FPP-RGD_2_. After mice placed in an abdominal position on the PET scanner gantry, approximately 7–12 MBq ^18^F-FPP-RGD_2_ were continuously injected (0.2 mL/30 s) into the tail vein. PET scans were started immediately after ^18^F-FPP-RGD_2_ injection was started. To confirm the^18^F-FPP-RGD_2_ binding to the integrin αvβ3, blockade experiments were performed by the co-injection of 60 μg c(RGDfK) with ^18^F-FPP-RGD_2_ in three mice. Dynamic data acquisition was performed for 90 min. After the PET scans, CT scans were performed to acquire anatomical information and to obtain data for the attenuation collection of PET images. The CT images were reconstructed using the filtered back-projection method (512 slices) and acquired PET images were reconstructed by the 3D-MLEM method with CT-based attenuation correction. Dynamic images (6 × 10 s, 4 × 1 min, 11 × 5 min, 3 × 10 min) for a time activity curve (TAC) were reconstructed. CT and PET images were automatically fused and analyzed by PMOD v3.6 (PMOD Technologies Ltd., Zürich, Switzerland). To calculate hepatic SUV 100 mm^3^ elliptic two regions-of-interest (ROI) were chosen excluding the aorta on the liver tissue were analyzed. Time 10–20 s PET image and CT image were used to set ROI in order to avoid the aorta on the liver. In addition, to clarify the influence of CDAHFD diet on systemic exposure, left ventricle of the heart instead of analysis of blood radioactivity was set as ROI for input analysis. TACs of liver and left ventricle were decay-corrected to the injection time and expressed as the standardized uptake value (SUV), where SUV = tissue radioactivity concentration (MBq/cm^3^)/injected radioactivity (MBq) × body weight (g). After the PET/CT scan, each mouse was euthanized and the plasma and liver were collected. Plasma and liver were immediately frozen in liquid nitrogen and stored at − 80 °C until protein assay. Liver was also collected and fixed by 10% formalin for histology. For quantitative RT-PCR, parts of livers were collected in RNAlater stabilization solution and stored at − 80 °C after 24 h stored at 4 °C.

### Western blotting

The hepatic amount of integrin αv and β3 subunit s protein was determined by western blot analysis, as described previously [24]. Briefly, liver homogenates were prepared, and 15 μg of protein was separated by electrophoresis on 4–12% gradient polyacrylamide gels. After transfer to a polyvinylidene fluoride membrane, they were blocked by 5% BSA buffer and incubated overnight at 4 °C with antibodies to mouse anti-integrin αv (1:100; BD Biosciences), rabbit anti-integrin β3 (1:1000; Abcam) and GAPDH (1:5000; Cell Signaling). After washing, the membrane was incubated with horseradish peroxidase-conjugated secondary antibodies and detected by LAS-3000.

### Quantitative RT-PCR analysis

The hepatic messenger RNA (mRNA) of integrin αv and β3 subunits, α-smooth muscle actin (α-SMA), and collagen type 1 alpha 1 (col1a1) was analyzed by RT-PCR as described previously [25]. Briefly, gene expression was measured using the 7500 Real-Time PCR System and Power SYBR™ Green PCR Master Mix (Applied Biosystems, CA, USA). The amplification method used 50 cycles of 95 °C for 5 min, 95 °C for 10 s, and 65 °C for 30 s. The 2-ΔΔCT method was used to calculate the relative mRNA expression normalized to 18S ribosomal RNA. The PCR primer sequences were as follows: Integrin αv-F: 5′-TCGTTTCTATCCCACCGCAG-3′. Integrin αv-R: 5′-TCGTTTCTATCCCACCGCAG-3′. Integrin β3-F: 5′-AGTGGCCGGGACAACTCT-3′, Integrin β3-R: 5′-AGACAAAGTCTCATCTGAGCACCA-3′. α-SMA-F: 5′-GAGCATCCGACACTGCTGACA-3′, α-SMA-R: 5′-AGCACAGCCTGAATAGCCACATAC-3′. Col1a1-F: 5′-GAGCGGAGAGTACTGGATCG-3′, Col1a1-R: 5′-TACTCGAACGGGAATCCATC-3′. 18S-F: 5′-CGGCTACCACATCCAAGGAA-3′, 18S-R: 5′-GCTGGAATTACCGCGCCT-3′.

### Statistical analysis

Quantitative data were expressed as the mean ± SD. Means were compared using Steel-Dwass test or Wilcoxon test. Spearman’s ranked correlation test was performed for evaluation of the correlation between protein expression of integrin αvβ3 and 80–90 min liver SUV of ^18^F-FPP-RGD_2_. *p* values < 0.05 were considered statistically significant.

## Results

### Blood biochemistry and liver histopathology in CDAHFD-fed mice

**Fig. 1:**
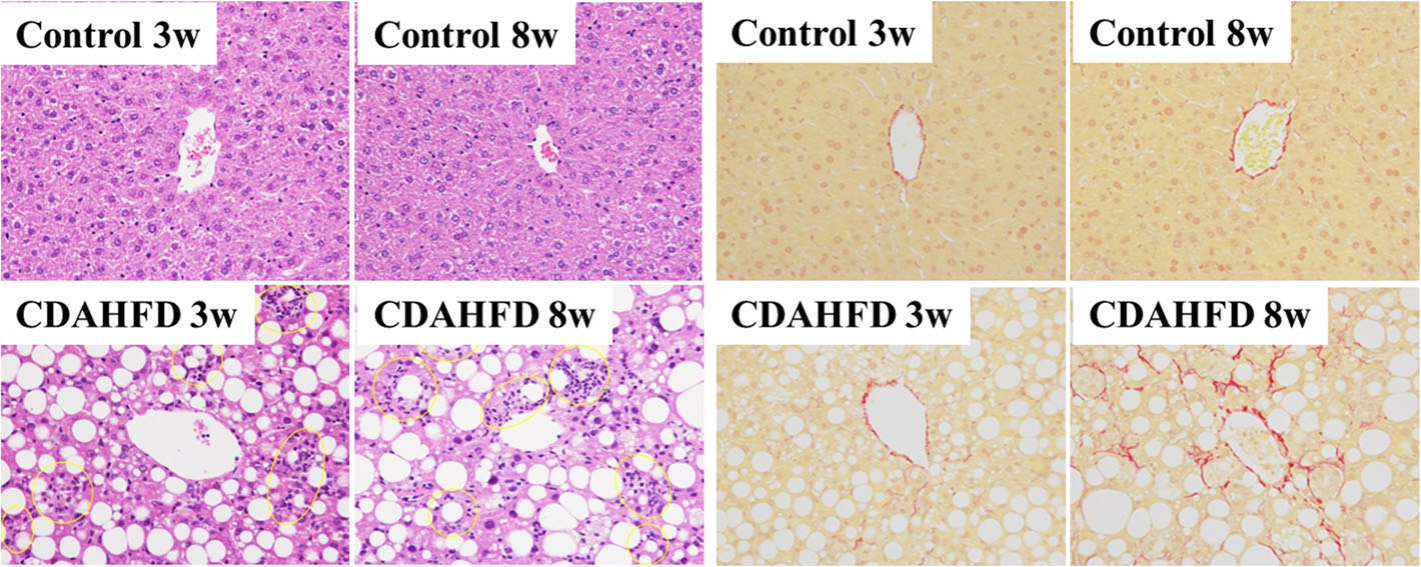
Hepatic histopathology in mice fed a control or choline-deficient, l-amino acid-defined, high-fat diet (CDAHFD) for 3 or 8 weeks. Representative photomicrographs of hepatic histology stained with hematoxylin and eosin (H&E) (left) and Sirius red (right) (× 200 magnification)

Plasma ALT and AST levels were significantly higher in 3- and 8-week CDAHFD-fed mice compared with respective control mice (ALT 19.20 ± 3.45 vs 772.10 ± 128.63, *p* < 0.01; 31.90 ± 16.52 vs 584.6 ± 475.74, *p* < 0.01, respectively. AST 32.80 ± 3.83 vs 436.6 ± 76.35, *p* < 0.01; 41.20 ± 12.69 vs 402.70 ± 242.78, *p* < 0.01, respectively) (Table 1). Histological analysis revealed that mice fed a CDAHFD for 3 and 8 weeks showed moderate-to-marked steatosis and inflammation was observed (Table 2, Fig. 1). No fibrotic areas were observed in mice fed the CDAHFD for 3 weeks but were observed in those with CDAHFD for 8 weeks (0.47 ± 0.19 vs 3.89 ± 1.81, *p* < 0.01) (Table 2, Fig. 1).

**Table 1:** Body, liver weight, and blood parameters in mice fed a CDAHFD

Parameter	Control 3 weeks	CDAHFD 3 weeks	Control 8 weeks	CDAHFD 8 weeks
Body weight (g)	24.44 ± 0.91	20.81 ± 0.96**	26.41 ± 1.56	21.66 ± 2.23**
Liver weight (g)	1.11 ± 0.11	1.47 ± 0.17**	1.12 ± 0.13	1.69 ± 0.24**
AST (IU/L)	32.80 ± 3.83	436.6 ± 76.35**	41.20 ± 12.69	402.70 ± 242.78**
ALT (IU/L)	19.20 ± 3.45	772.10 ± 128.63**	31.90 ± 16.52	584.6 ± 475.74**
TG (mg/dL)	155.7 ± 48.57	41.50 ± 17.69**	146.80 ± 65.67	21.50 ± 3.54**
TC (mg/dL)	80.40 ± 7.91	49.00 ± 6.95**	79.70 ± 9.50	36.3 ± 4.14**

Statistical differences were assessed using Steel-Dwass test

*AST* aspartate transaminase, *ALT* alanine aminotransferase, *TC* total cholesterol, *TG*, triglyceride

**p < 0.01 compared with respective control mice

**Table 2:** Histological analysis of the liver in mice fed a CDAHFD

Parameter	Control 3 weeks	CDAHFD 3 weeks	Control 8 weeks	CDAHFD 8 weeks
Steatosis score	0.00 ± 0.00	1.75 ± 0.71**	0.00 ± 0.00	1.88 ± 0.35**
Inflammation score	0.00 ± 0.00	2.88 ± 0.35**	0.00 ± 0.00	3.00 ± 0.00**
Ballooning score	0.00 ± 0.00	0.38 ± 0.52**	0.00 ± 0.00	0.88 ± 0.64**
NAFLD activity score	0.00 ± 0.00	5.00 ± 1.07**	0.00 ± 0.00	5.75 ± 0.71**
Fibrosis area (%)	0.61 ± 0.29	0.47 ± 0.19	0.65 ± 0.18	3.89 ± 1.81**^##^

Representative photomicrographs of hepatic histology stained with hematoxylin and eosin (H&E) and Sirius red. Steatosis and inflammation scores ranged from 0 to 3 (normal = 0; minimal = 1; moderate = 2; marked = 3). Ballooning score ranged from 0 to 2 (normal = 0; minimal = 1; marked = 2). NAFLD activity score (NAS) was calculated by using the sum of each histological score. Data are expressed as the mean ± SD (*n* = 8 mice per group). Statistical differences were assessed using Steel-Dwass test. Hematoxylin and eosin (H&E) and Sirius red, × 200 magnification

*p < 0.05, **p < 0.01 compared with respective control mice. ^##^*p* < 0.01 compared with CDAHFD 3 weeks

### ^18^F-RPP-RGD_2_ PET imaging in CDAHFD-fed mice

**Fig. 2:**
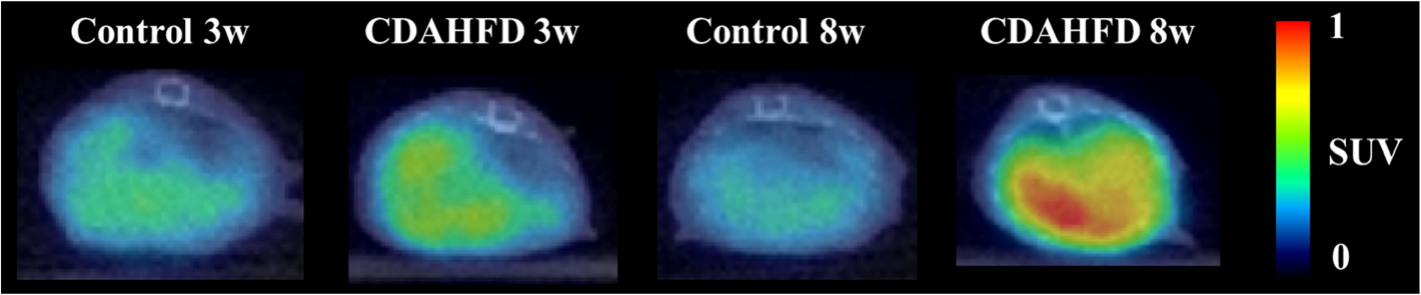
Representative PET/CT fusion images in the livers of mice fed a control or choline-deficient, l-amino acid-defined, high-fat diet (CDAHFD) at 80–90 min

**Fig. 3:**
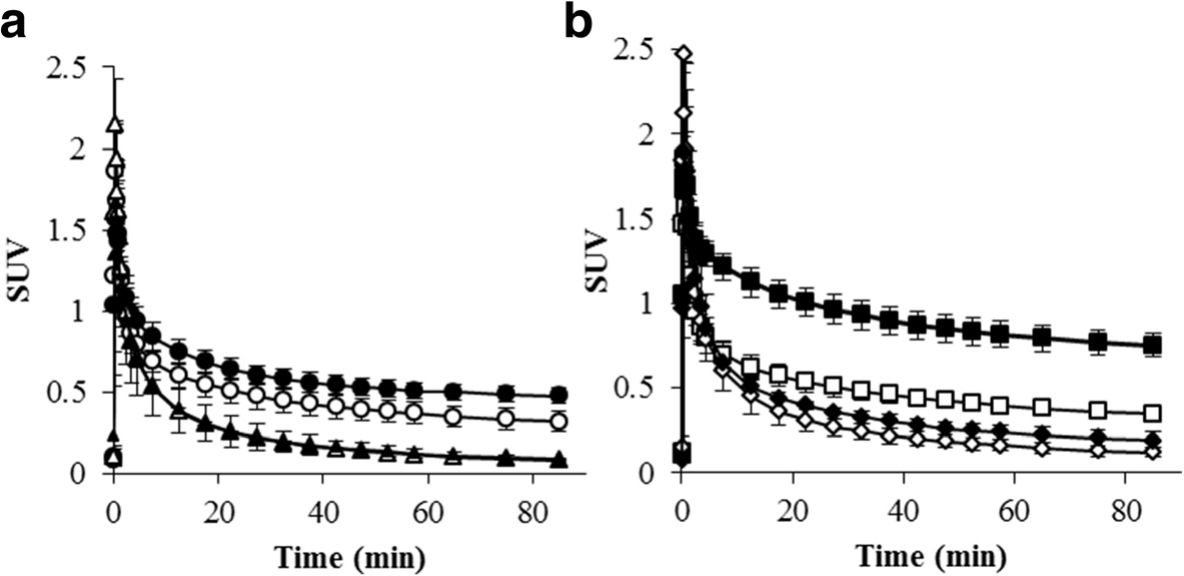
Hepatic time activity curves after ^18^F-FPP-RGD_2_ injection in mice fed a control (**a**) or methionine choline-deficient, l-amino acid-defined, high-fat diet (CDAHFD) (**b**) (*n* = 5 per group). Sixty micrograms of c(RGDfK) was co-injected with ^18^F-FPP-RGD_2_ into each group for the blockade study (*n* = 3 per group). white circle, control 3 weeks; black circle, CDAHFD 3 weeks; white triangle, control 3 weeks + cRGDfK; black triangle, CDAHFD 3 weeks + cRGDfK; white square, control 8 weeks; black square, CDAHFD 8 weeks; white diamond, control 8 weeks + cRGDfK; and black diamond, CDAHFD 8 weeks + cRGDfK

**Fig. 4:**
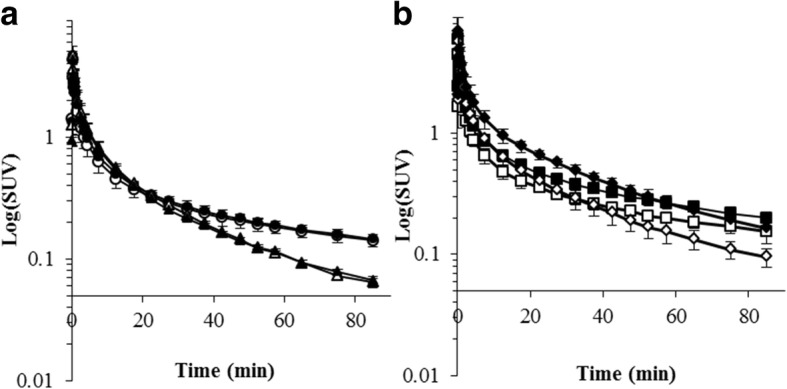
Left ventricle time activity curves after ^18^F-FPP-RGD_2_ injection in mice fed a control (**a**) or methionine choline-deficient, l-amino acid-defined, high-fat diet (CDAHFD) (**b**) (*n* = 5 per group). Sixty micrograms of c(RGDfK) was co-injected with ^18^F-FPP-RGD_2_ into each group for the blockade study (*n* = 3 per group). white circle, control 3 weeks; black circle, CDAHFD 3 weeks; white triangle, control 3 weeks + cRGDfK; black triangle, CDAHFD 3 weeks + cRGDfK; white square, control 8 weeks; black square, CDAHFD 8 weeks; white diamond, control 8 weeks + cRGDfK; and black diamond, CDAHFD 8 weeks + cRGDfK

**Fig. 5:**
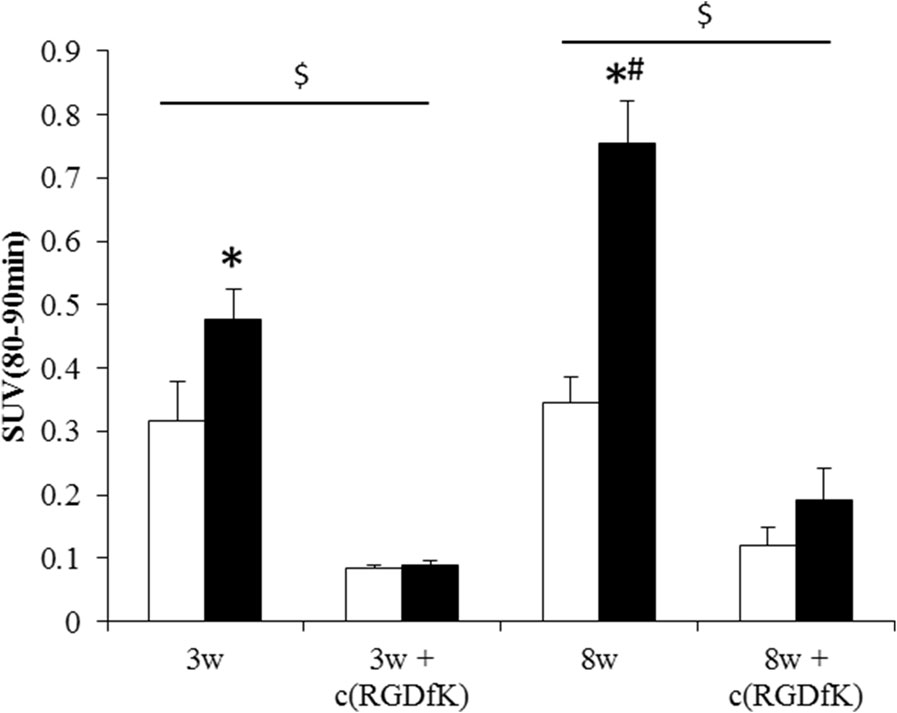
Hepatic SUV at 80–90 min. Data are expressed as the mean ± SD (*n* = 5 per group or *n* = 3 per group (+cRGDfK)). Statistical differences were assessed using Steel-Dwass test; *p < 0.05, compared with respective control mice, ^#^*p* < 0.05 compared with mice fed the CDAHFD for 3 weeks and Wilcoxon test, ^$^*p* < 0.05 compared with respective blockade group. White bar, control group; black bar, CDAHFD group

PET images of ^18^F-FPP-RGD_2_ at 80–90 min and time activity curves (TACs) of the liver and heart, mainly the covered left ventricle, are shown in Figs. 2, 3, and 4. Higher uptake of ^18^F-FPP-RGD_2_ was observed in mice fed the CDAHFD for 3 and 8 weeks compared with control mice. Hepatic TACs revealed that the clearance of ^18^F-FPP-RGD_2_ in CDAHFD-fed mice, which was calculated using the following equation: ((SUV_0–5 min_ − SUV_80–90 min_)/SUV_0–5 min_), was slower than that of respective control mice (control 3 weeks vs CDAHFD 3 weeks = 0.69 vs 0.56, control 8 weeks vs CDAHFD 8 weeks = 0.66 vs 0.45). ^18^F-FPP-RGD_2_ uptake in the heart was highest at 20 s and was eliminated rapidly from all groups (Fig. 4a, b). Hepatic radioactivity of excess cold-c(RGDfK) co-injection groups were rapidly cleared from the liver (Fig. 3a, b). At 80–90 min, the SUV of mice fed the CDAHFD at 3 and 8 weeks was significantly higher than that of respective control mice (0.32 ± 0.06 vs 0.48 ± 0.05, *p* < 0.05, 0.35 ± 0.04 vs 0.75 ± 0.07, *p* < 0.05) (Fig. 5). In the blockade study, all groups had accelerated liver clearance of ^18^F-FPP-RGD_2_ and decreased SUV at 80–90 min compared with the respective control groups.

### Hepatic expression of integrin αv and β3 in CDAHFD-fed mice

**Fig. 6:**
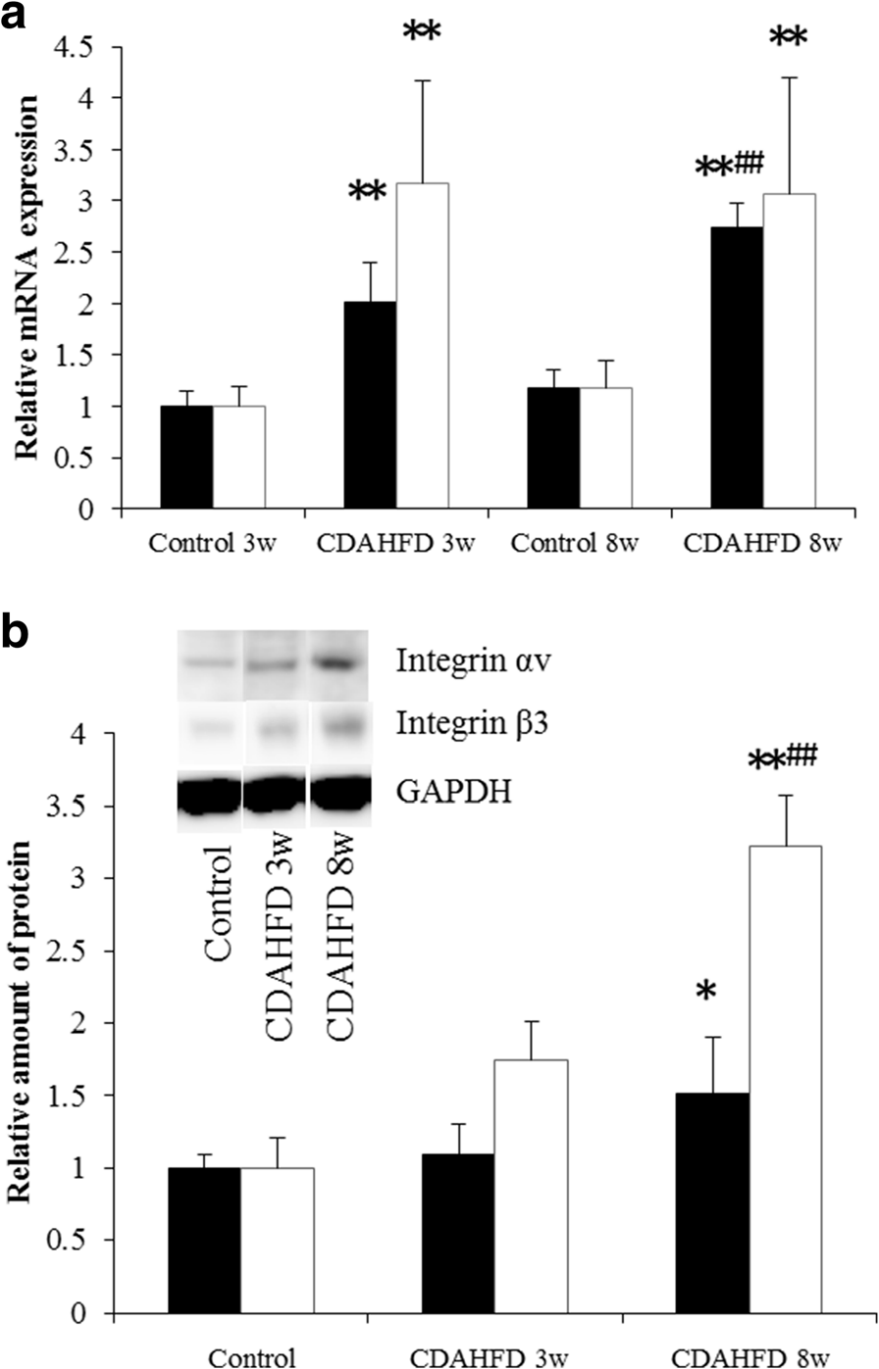
Hepatic mRNA (**a**) and protein levels (**b**) of integrin αv and β3 subunits in the livers of control and choline-deficient, l-amino acid-defined, high-fat diet (CDAHFD)-fed mice. Data are expressed as the mean ± SD (*n* = 8 mice per group). Statistical differences were assessed using Steel-Dwass test. *p < 0.05, **p < 0.01 compared with respective control mice. ^##^*p* < 0.01, compared with the CDAHFD 3 weeks group. White bar, integrin αv; black bar, integrin β3

**Fig. 7:**
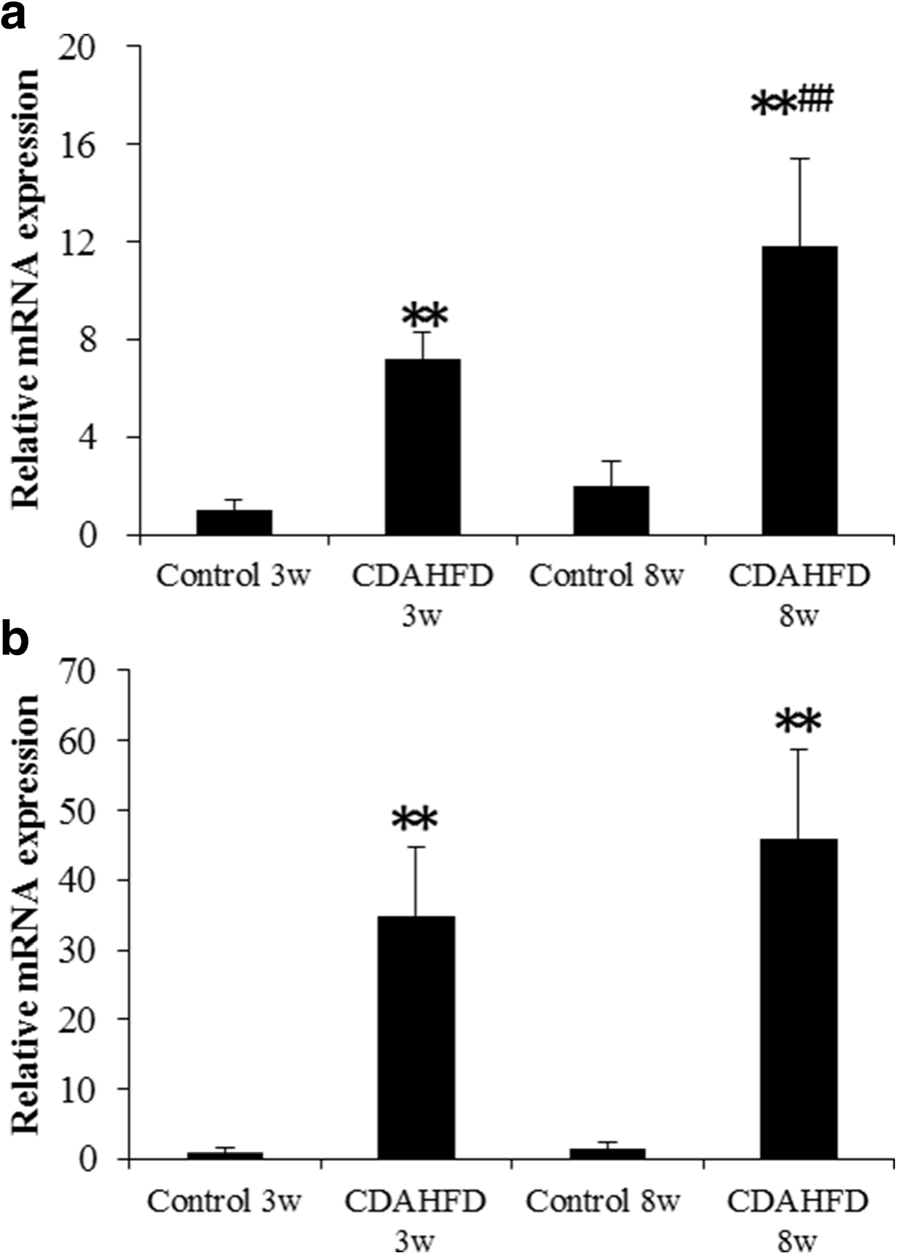
Hepatic mRNA levels of **a** α-smooth muscle actin (α-SMA) and **b** collagen type 1a1 (Col1a1) in the livers of mice fed a control or choline-deficient, l-amino acid-defined, high-fat diet (CDAHFD). Data are expressed as the mean ± SD (*n* = 8 mice per group). Statistical differences were assessed using Steel-Dwass test. **p < 0.01 compared with respective control mice. ^##^*p* < 0.01, compared with the CDAHFD 3 weeks group

Hepatic mRNA and protein levels of integrin αv and β3 were increased by prolonged CDAHFD (Fig. 6). Mice fed the CDAHFD for 8 weeks had the highest protein and mRNA expression of all groups. Hepatic α-SMA and Col1a1 mRNA expressions were also markedly increased in 3- and 8-week CDAHFD-fed mice (Fig. 7).

### Correlation of hepatic uptake of ^18^F-FPP-RGD_2_ and protein expression of integrin αv or β3, or Sirius red-positive area in CDAHFD-fed mice

**Fig. 8:**
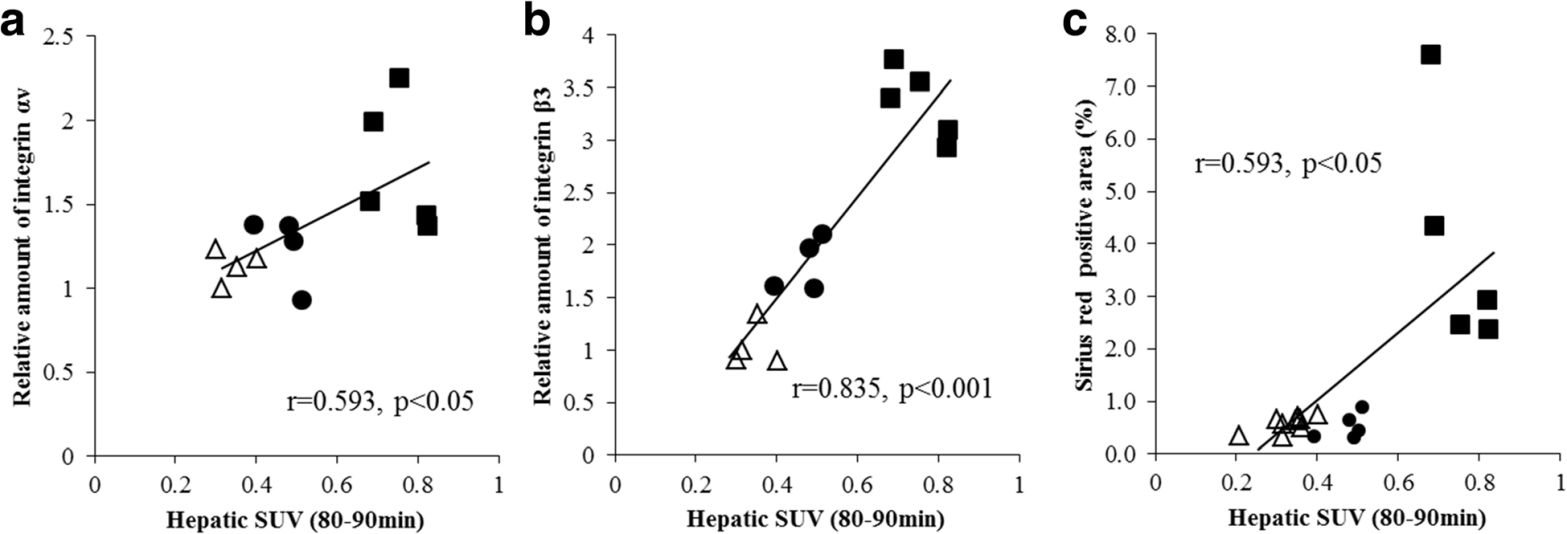
Correlation between hepatic ^18^F-FPP-RGD_2_ uptake and integrin αv (**a**), β3 (**b**) subunits, and Sirius red-positive area (**c**). Correlations were analyzed using Spearman’s ranked correlation test. white triangle, control; black circle, CDAHFD 3 weeks; black square,CDAHFD 8 weeks

We evaluated the correlation between the hepatic uptake of ^18^F-FPP-RGD_2_ SUV at 80–90 min and protein expression of integrin αv or β3. Liver SUV at 80–90 min showed a positive correlation with integrin αv or β3 (*r* = 0.593, *p* < 0.05 and *r* = 0.835, *p* < 0.001) (Fig. 8a, b). We also evaluated correlation between the hepatic of ^18^F-FPP-RGD_2_ SUV at 80–90 min and Sirius red-positive area. Liver SUV at 80–90 min showed a positive correlation with Sirius red-positive area (*r* = 0.593, *p* < 0.05) (Fig. 8c).

## Discussion

In this study, we clearly showed that the hepatic uptake of ^18^F-FPP-RGD_2_ was correlated with the hepatic expression of integrin αvβ3 in CDAHFD-fed NASH model mice. The CDAHFD-fed model was developed as a new NAFLD/NASH mouse model and has a rapid onset and progression of hepatic fibrosis compared with the methionine-choline-deficient diet-fed mouse model [20]. In the present study, high ALT and ASL levels, steatosis, inflammation, and ballooning were observed in 3- and 8-week CDAHFD-fed mice. To assess the NASH pathology, the sum of steatosis score, inflammation score, and ballooning score called NAS, were used. According to the criteria, a NAS of 5 or more is diagnosed as “definitive NASH” and NAS of 3 or 4 is diagnosed as ‘borderline NASH’ [26]. Therefore, in this study, CDAHFD 3 and 8 weeks fed mice were considered as NASH-like pathology. However, Sirius red stain-positive areas were only observed in CDAHFD mice fed for 8 weeks. These results indicate that CDAHFD mice fed for 3 weeks developed NAFLD/NASH with minimal or no fibrosis and that CDAHFD mice fed for 8 weeks developed moderate fibrosis. A previous study reported that the mRNA and protein expressions of integrin αvβ3 were increased during the development and progression of liver fibrosis in CCl_4_ and thioacetamide models, which are used as hepatic fibrosis models commonly [19, 20, 27]. In this study, CDAHFD mice fed for 3 weeks had increased hepatic integrin αv and β3 and α-SMA mRNA expressions, which are indicators of HSCs. Mouse fed a methionine choline-deficient diet, another commonly used NASH model, revealed an increase in α-SMA before fibrosis [21]. Therefore, it was considered that integrin αvβ3 might be increased with HSC activation rather than fibrosis in the livers of NASH model mice induced by a methionine choline-deficient diet.

^18^F-FPP-RGD_2_ is a commonly used ^18^F labeled RGD PET probe in clinical and non-clinical studies of tumors that express integrin αvβ3 [17, 28]. To our knowledge, this is the first study to evaluate the relationship between the hepatic uptake of ^18^F-FPP-RGD_2_ and integrin αvβ3 expression using PET in CDAHFD-fed NASH/NAFLD model mice. In a previous study, Li et al. reported that integrin αvβ3 was co-localized with α-SMA positive areas by immunofluorescence and that fluorescent labeled RGD bound to activate HSCs [19]. Caiyuan et al. also reported that integrin β3 was co-localized with α-SMA and that MRI contrast agent labeled RGD accumulated in HSCs [20] indicating ^18^F-FPP-RGD_2_ also specifically bound to integrin αvβ3 on HSCs. In a PET imaging study, the hepatic uptake of ^18^F-FPP-RGD_2_ was increased with CDAHFD feeding and excess unlabeled cRGD co-injection reduced ^18^F-FPP-RGD_2_ accumulation in all groups. These results indicated that ^18^F-FPP-RGD_2_ bound to integrin αvβ3 in vivo. The hepatic uptake (SUV) of ^18^F-FPP-RGD_2_ remained constant from 30 to 90 min in both control and model mice. On the other hand, the liver ratio to the heart of ^18^F-FPP-RGD_2_ uptake was maximum at 90 min, suggesting the minimum effects of blood radioactivity to tissue radioactivity. Therefore, we used this time point for the SUV values in all analyses. At 80–90 min, the hepatic SUV of ^18^F-FPP-RGD_2_ in mice fed the CDAHFD for 3 and 8 weeks was increased compared with the respective control mice and the highest uptake was in mice fed the CDAHFD for 8 weeks. A previous SPECT scintigraphy study using ^99m^Tc-labeled cRGD in a thioacetamide treated rat model reported that the radioactivity of liver-to-heart ratio was increased with integrin αvβ3 expression and fibrosis stage [19]. On the other hand, there was no correlation study between SPECT and integrin αvβ3 expression or fibrosis stage using the same animals. In the present study, the hepatic SUV of ^18^F-FPP-RGD_2_ well correlated with integrin αv and β3 protein expression. The hepatic SUV of ^18^F-FPP-RGD_2_ also showed correlation with Sirius red-positive area. On the other hand, in CDAHFD for 3 weeks fed mice, hepatic SUV of ^18^F-FPP-RGD_2_ was increased before fibrosis. While integrin αv is expressed by parenchymal cells and HSCs, most integrin β3 was expressed on HSCs [29]. Furthermore, while integrin αv forms complexes with several β subunits (β1, β3, β5, β6, β8), integrin β3 forms complexes with only two type α subunits, αv and αIIb [30]. It might be thought that amount of integrin αvβ3 is similar to integrin β3 rather than integrin αv. Therefore, hepatic SUV of ^18^F-FPP-RGD_2_ might be correlated with integrin β3rather than integrin αv and Sirius red-positive area. These results indicate that ^18^F-FPP-RGD_2_ PET has the potential to evaluate the expression of integrin αvβ3 on hepatic cells including activated HSCs and might have potential to predict fibrosis. Furthermore, ^18^F-FPP-RGD_2_ PET might be helpful for development of anti-fibrotic agent and decision about therapeutic intervention at early fibrosis stage in NASH patient. Steatosis and inflammation score are used to evaluate NAFLD activity score, which discriminates NASH from simple steatosis. However, steatosis and inflammation were severe without any significant difference between mice fed the CDAHFD for 3 and 8 weeks. Therefore, further studies are needed to prove that ^18^F-FPP-RGD_2_ PET could evaluate NASH pathology.

In quantitative PET imaging study, it is important to evaluate input function. Because of the small size of mice, arterial input function through blood sampling is technically challenging and we did not collect the arterial blood. A previous study used radioactivity of the left ventricle from a PET image as the image-derived input function [31]. In the present study, the radioactivity of the heart, mainly including the left ventricle increased immediately after injection and then decreased rapidly. Therefore, most of the radioactivity in the heart would be thought as image-derived input function. In mice fed the CDAHFD for 3 weeks, image-derived input function was not changed significantly. On the other hand, in mice fed the CDAHFD for 8 weeks, although there was no change of image-derived input function at early phase (about 0–5 min), a slight increase was observed at late phase. These data indicate that increased liver uptake of ^18^F-FPP-RGD_2_ partly might be due to increased input function in mice given CDAHFD for 8 weeks. Because mouse ventricle was small, it is difficult to exclude the cardiac muscle completely in setting the region of interest. Then, although left ventricle ROI set carefully, image-derived input function might include the spillover of radioactivity in cardiac muscle. Further studies, especially correct input function evaluation, will be needed for assessing the quantitative analysis of the hepatic uptake of ^18^F-FPP-RGD_2_ in small animal.

## Conclusions

PET imaging showed ^18^F-FPP-RGD_2_ uptake was increased before the onset of fibrosis and correlated with integrin αvβ3 expression, especially β3 expression. ^18^F-FPP-RGD_2_ PET imaging might be useful to non-invasively predict the fibrosis risk in NASH patients.
